# The association of Metabolic Syndrome and its Components with the Incidence and Survival of Colorectal Cancer: A Systematic Review and Meta-analysis

**DOI:** 10.7150/ijbs.52452

**Published:** 2021-01-01

**Authors:** Fei Han, Guanghai Wu, Shuai Zhang, Judong Zhang, Yongjie Zhao, Jing Xu

**Affiliations:** 1 NHC Key Laboratory of Hormones and Development, Tianjin Key Laboratory of Metabolic Diseases, Chu Hsien-I Memorial Hospital & Tianjin Institute of Endocrinology, Tianjin Medical University, Tianjin 300134, China.; 2 Department of General Surgery, Tianjin Union Medical Center, Tianjin 300121, China.

**Keywords:** metabolic syndrome, colorectal cancer, incidence, survival, meta-analysis

## Abstract

**Background:** This meta-analysis was aimed to quantitatively assess the associations of metabolic syndrome (MetS) and its components with colorectal cancer (CRC).

**Methods:** PubMed, EMBASE and Web of Science databases were systematically searched for eligible studies. A total of 18 studies for CRC incidence and 12 studies for CRC mortality were identified.

**Results:** MetS was associated with an increased risk of CRC incidence and mortality in male (RR: 1.28, 95 % CI 1.16-1.39, and 1.24, 1.18-1.31, respectively) and correlated with an increased risk of CRC incidence in female (RR: 1.21, 1.13-1.30), but not with CRC mortality in female. MetS increased the risk of cancer-specific mortality (RR: 1.72, 1.03-2.42), but not overall mortality. The risk estimates of CRC incidence changed little depending on age, sex, cancer site, the type of studies, ethnicity, publication year, or definition of MetS. As for CRC mortality, further stratified analyses indicated statistical significance in studies with assessing cancer-specific survival outcome, in male, a cohort design, ethnicity of non-Chinese or with definition of MetS as ≥ 3 metabolic abnormalities. Obesity and hyperglycemia are risk factors of CRC incidence in both male and female. Only dysglycemia is the risk factor for CRC mortality.

**Conclusions:** MetS is associated with an increased risk of CRC incidence and cancer-specific mortality, but not overall mortality. In addition, MetS may increase the CRC mortality in male rather than in female. However, since most of the studies on CRC mortality were conducted in Chinese, further studies are needed to clarify this connection.

## Introduction

Metabolic syndrome (MetS) is a cluster of metabolic risk factors that includes abdominal obesity, hypertension, hyperglycemia, and dyslipidemia 1. The prevalence of MetS ranges between 34.8% and 41.9% in the US and 18% and 46% in Europe 2, 3. The number varies depending on race, environmental factors, genetic differences, physical activity level, eating habits, and differences in measurement standards 4. However, the rapid growing of MetS prevalence raises lots of public health concerns including cancer.

Nowadays, given the rising prevalence of MetS all over the world and the high occurrence of cancers, especially colorectal and breast cancers, many cases of cancer may be linked to MetS 5, 6. MetS and cancer share many modifiable risk factors including age, genetic factors, obesity, physical inactivity, unhealthy diet, alcohol and smoking 7. MetS has been closely linked to cancer, as it increases cancer risk and cancer-related mortality. Moreover, MetS usually occurs as a consequence of specific chemotherapy drugs and radiotherapy; therefore, MetS and diabetes mellitus have been increasingly recognized as long-term complications of childhood cancer treatment 8. Hence, cancer survivors have an increased risk of MetS 7. So, clarifying the underlying mechanisms linked MetS to cancer is important to prevent or delay these two conditions.

Colorectal cancer (CRC) is the third most commonly diagnosed cancer worldwide and one of the leading causes of cancer-specific death 9, with more than 1.1 million cancer deaths expected by 2030 10. According to the published studies, diet 11, obesity 12, alcohol intake 13 and diabetes 14 are all risk factors for the occurrence and mortality in patients with CRC. Since 2001, several epidemiological studies have investigated the association between MetS and CRC risk but showed inconsistent results. As some studies showed that, MetS plays an important role in CRC 15-17. However, there were also studies showed no significant correlation between the two 18-22. In the above studies, MetS may increase the risk of CRC in male rather than female 18, 20. In addition, the correlation may also differ because of cancer site 23. Apart from increasing the cancer risk, studies also reported that MetS is an important risk factor for cancer mortality. It is reported that patients with MetS has a higher mortality 24, 25. However, some studies declared that MetS has no effect on CRC mortality 26, 27. Many studies also discussed the role of different MetS components in the development and progression of CRC. Most of them showed that obesity and diabetes may be risk factors for CRC 19, 25, while some are not 21, 27.

In view of the ambivalent results listed above, clarifying the association of MetS and its components with the incidence and survival of colorectal cancer, may help revealing important risk and prognostic factors of CRC. Based on the results, clinicians can also make effective strategies to prevent the onset and development of CRC. Esposito et al. carried out a meta-analysis to unearth the correlation between MetS and its components with the progression of CRC seven years ago 28, however, a lot of new clinical research were conducted after that. Due to the small numbers of studies and patients included, this previous review might not fully explore the potential variation of this association. As data on the relation between MetS and CRC occurrence and survival are accumulating lately, we therefore decided to synthesize the results of published studies to test whether MetS and its individual components can predict risk and outcomes in patients with CRC.

## Methods

### Search strategy

PubMed, EMBASE databases and Web of Science were searched from inception to May 31st, 2020, for eligible studies on the relationship between MetS and CRC. The terms used to retrieve literatures were the following: Colorectal OR colon OR rectal AND cancer OR carcinoma OR malignancy OR tumor OR neoplasm AND metabolic syndrome AND risk OR incidence OR survival OR prognosis OR mortality. We also referred to the reference lists from reviews or relevant papers to get more eligible researches. Conference abstracts were also included if sufficient data were provided. There was no language restriction. Two authors independently performed the literature search and identified potential studies of the title, abstract and full-text.

### Selection criteria

Reports were included if they met the criteria as follows: (1) study designs: case-control studies, cohort studies and randomized controlled trials (RCTs); (2) risk estimates of CRC incidence or mortality with 95% CIs (Confidences Intervals) were reported. If the same data were used in several studies, we selected the publication with the largest number of cases or more details. The exclusion criteria: (1) letters, editorials, abstracts, reviews, case reports or expert opinions; (2) studies not based on people; (3) outdated articles with little significance or credibility. Literature search also was independently done by two authors (F.H. and G.W.).

### Data Extraction

From each included study, data were independently extracted by two investigators (F.H. and G.W.) using a standardized data extraction form. Briefly, we recorded study characteristics including first author name, publication year, country, average/range of age, mean/median duration of follow-up, specific outcomes, total number of individuals, number of cases, and risk estimates and their 95 % CIs. In order to dissect the influence of any single component of the metabolic syndrome, risk estimates for each single component were collected. Disagreements between investigators were discussed and resolved by an additional reviewer.

### Quality Assessment

Study quality was independently assessed by two of us (F.H. and G.W.). Quality of the included studies was evaluated by use of the Newcastle Ottawa Scale (NOS). According to its criteria, studies were assessed on the basis of three perspectives: selection, comparability and outcomes. The full score was defined as 9 stars, and a study was classified as low, moderate and high-quality using 0-3, 4-6 and 7-9 stars, respectively. Differences were resolved by discussion. PRISMA (preferred reporting items for systematic reviews and meta-analyses) checklist was followed for reporting systematic reviews and meta-analyses 29.

### Statistical analysis

In a conservative approach, the random-effects estimates of relative risk (RR), which allow for variation of true effects across studies, were taken as “main results” 28. Statistical heterogeneity among studies was evaluated with the use of *I*^2^ statistic. Significant heterogeneity was assumed for *I*^2^ > 50% or a *Q* test* p*-value < 0.05 30. We utilized the random-effects model to combine RRs from single studies if obvious heterogeneity was observed 31. Subgroup analyses were conducted to explore potential sources of heterogeneity across studies. In the sensitivity analysis, studies were omitted one by one and the others were analyzed to evaluate the effect of a single study on the summary risk estimates. Publication bias was assessed statistically with Kendall's tau 32, 33. A* p*-value < 0.05 in these tests suggests the presence of publication bias. We utilized STATA (Version 12.0, College Station, TX, USA) to perform these analyses.

## Results

### Literature Search and Study Characteristics

**Figure 1:**
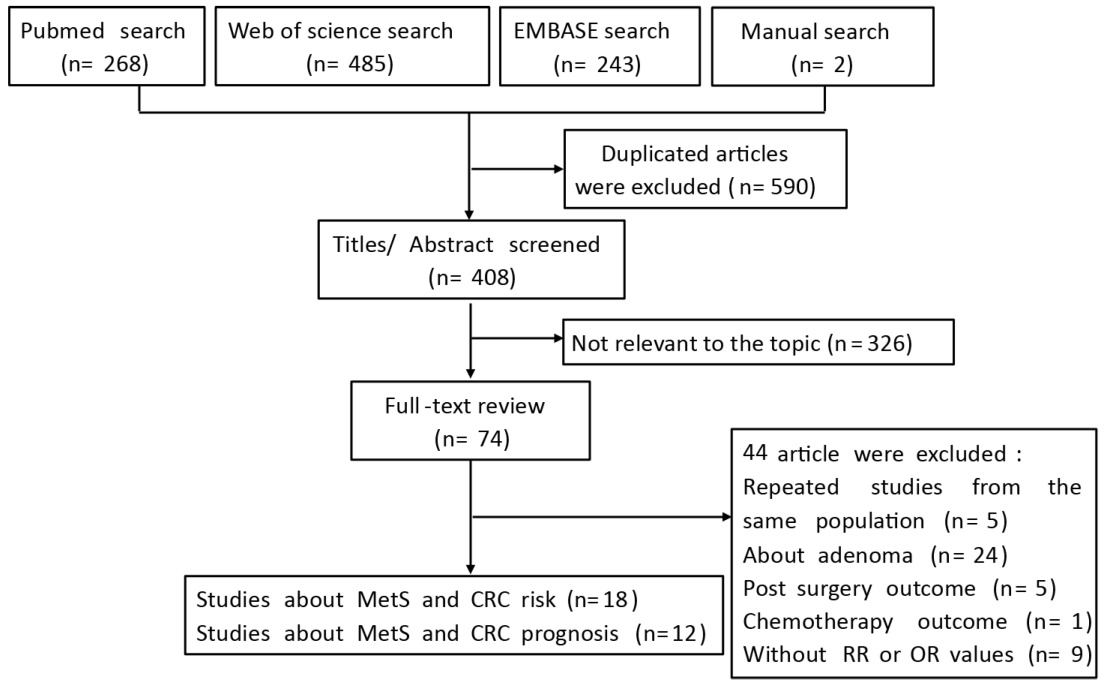
Flow diagram of the systematic literature process.

The flow chart of the literature search is presented in **Figure 1.** After a comprehensive search, a number of 998 citations were identified. Among these, 408 citations remained, after the exclusion of duplicates, and 326 citations were excluded by screening the titles and abstracts, leaving 74 potentially relevant articles for full-text examination. Of these, 44 citations were excluded, because they did not meet the inclusion criteria. Finally, 30 articles were included for data synthesis.

Basic information concerning the eligible studies are listed in **Tables 1 and 2.** From 2006 to 2019, a total of 10 prospective cohort studies 18-21, 25, 34-38, 6 case-control studies 39-44, one retrospective cohort study 22 and one cross-sectional study 45 were included in the meta-analysis of CRC incidence, and 8 prospective cohort studies 25-27, 46-49, 2 retrospective cohort study 50, 51 and 3 case-control studies 52-54 were included in the meta-analysis for CRC mortality. Thirteen studies were conducted in Asia 21, 22, 27, 37, 38, 42, 43, 46, 48, 50-52, 54, eight were performed in Europe 20, 25, 34, 39-41, 44, 45, and the remaining studies were executed in the USA 18, 19, 26, 35, 36, 47, 49. Most of them obeyed the traditional definition from IDF, ATP III or AHA, while some were not. Three studies were limited to males 19, 34, 38 and two to females 35, 36 in the meta-analysis of CRC incidence, and two studies were limited to males 26, 50 in the meta-analysis for CRC mortality. As for the article about CRC mortality, four studies investigated stage I-III patients 47, 49, 50, 52 and the remaining studies investigated stage I-IV patients. The level of covariate adjustment in the individual studies differed, most studies adjusted for age, sex, smoking and alcohol. On the basis of the NOS criteria, most studies were classified as high-quality, and the remaining studies were classified as moderate-quality 20, 22, 40, 42.

### Meta-Analysis for association between MetS and CRC

**Figure 2:**
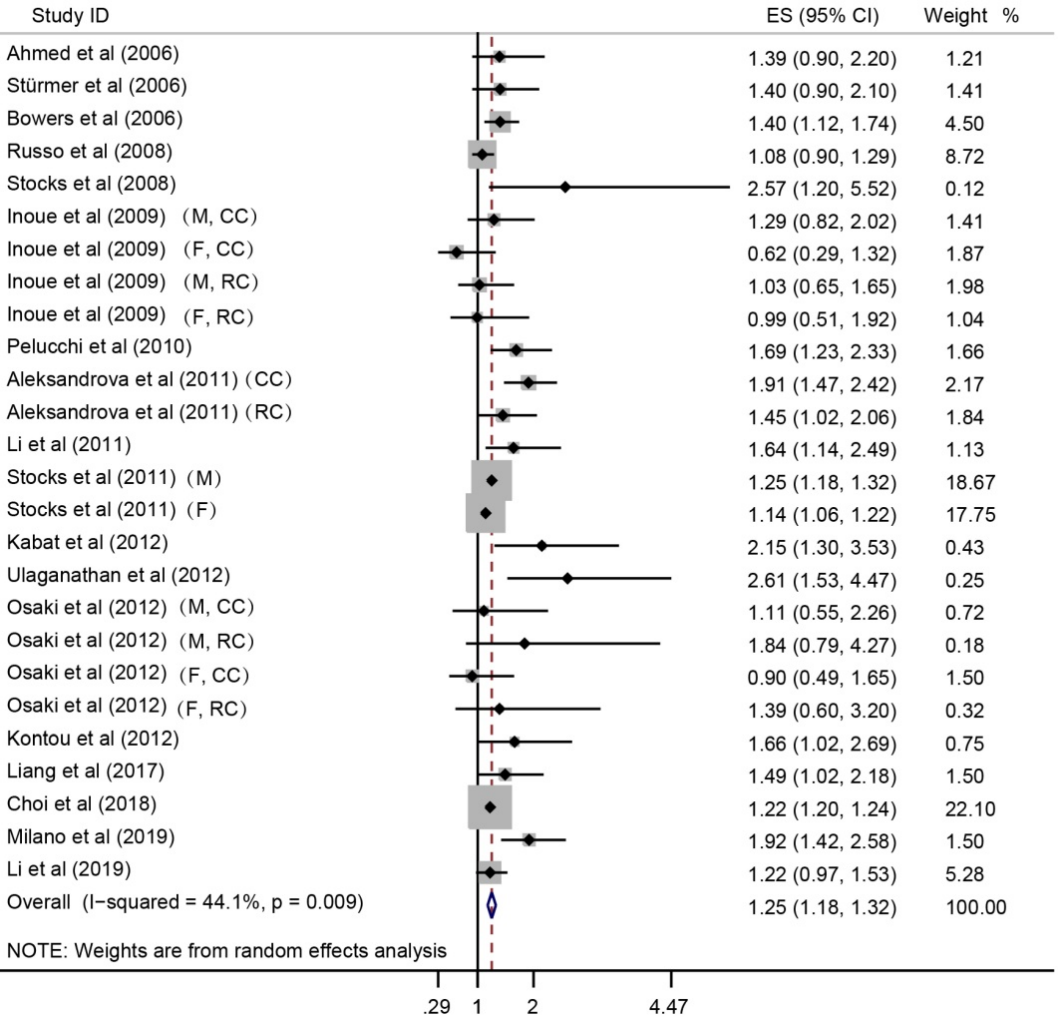
** Forest plot to quantify the association between MetS and CRC incidence.** ES, effect size; 95% CI, 95% confidence interval; M, male; F, female; CC, colon cancer; RC, rectal cancer.

**Figure 3:**
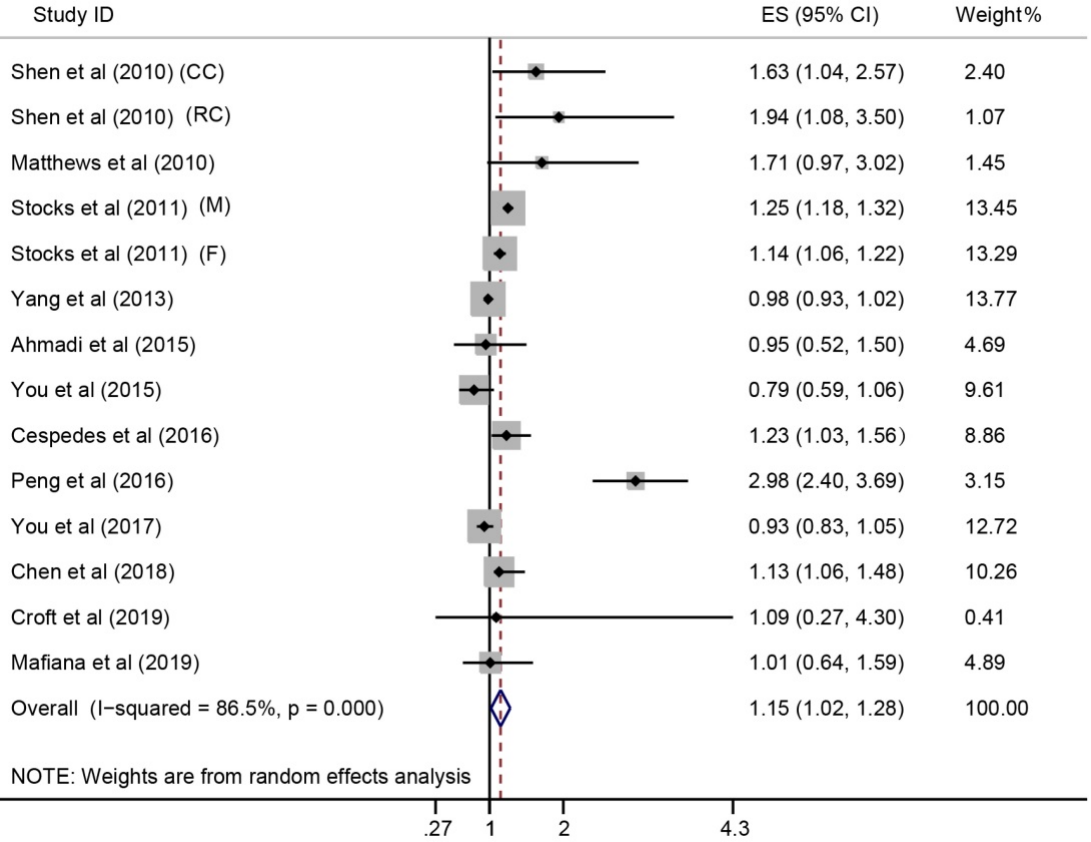
** Forest plot for the association between MetS and CRC survival.** ES, effect size; 95% CI, 95% confidence interval; M, male; F, female; CC, colon cancer; RC, rectal cancer.

In the meta-analysis combining results of the included studies, MetS was associated with CRC risk (Summary RR = 1.25; 95% CI: 1.18-1.32), with a between-study heterogeneity (Q (*df* = 25) = 44.7, *p*-value = 0.009; *I*^2^ = 44.1%) (**Figure 2**). In overall analysis, the presence of MetS was associated with a 15% increased mortality risk in CRC (Summary RR = 1.15; 95% CI: 1.02-1.28), and this association was obsessed by significant between-study heterogeneity (Q (*df* = 13) = 96.56, *p*-value = 0.000; *I*^2^ = 86.5%) (**Figure 3**).

### Subgroup Analysis

**Figure 4:**
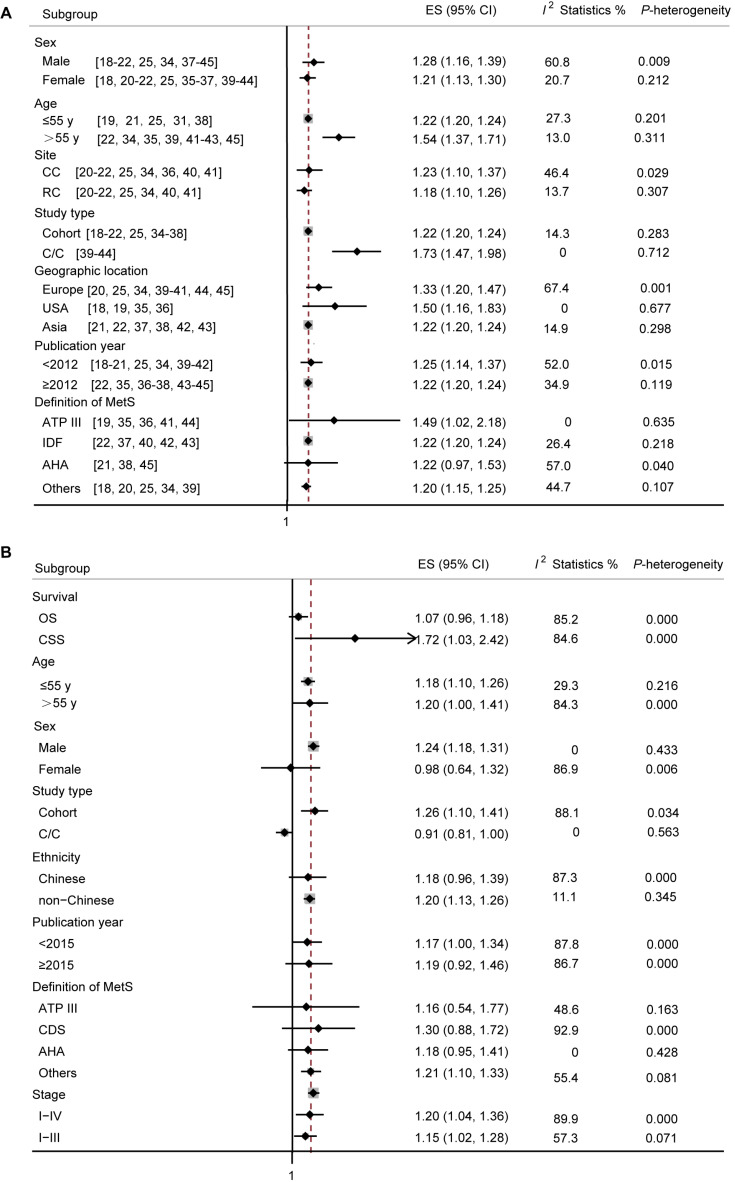
** Subgroup analyses for cancer incidence and survival in patients with CRC.** (A) Subgroup analyses for the association between MetS and CRC incidence. (B) Subgroup analyses for the association between MetS and survival in patients with CRC. ES, effect size; 95% CI, 95% confidence interval; IDF: International Diabetes Federation; AHA: American Heart Association; ATP III: Adult Treatment Panel III; CDS: Chinese Diabetes Society; OS: overall survival; CSS: cancer-specific survival.

In terms of total CRC incidence (**Figure 4A**), a RR of 1.28 (95% CI: 1.16-1.39) was found in male, with a between-study heterogeneity (*p*-value = 0.009; *I*^2^ = 60.8%). In articles reporting incidence of CRC in female patients with MetS, a RR of 1.21 (95 % CI: 1.13-1.30) was found. However, no significant heterogeneity among the studies was found (*p*-value = 0.212; *I*^2^ = 20.7%). Studies were divided according to average age of included patients, the older people with MetS suffer a higher risk of CRC. Studies were divided according to cancer site, and the colon cancer group showed an association between MetS and cancer risk (Pooled RR = 1.23; 95% CI: 1.10-1.37; *p*-value = 0.029; *I*^2^ = 46.4%), and also there was a significant correlation in the rectal cancer subgroup (Pooled RR = 1.18; 95% CI: 1.10-1.26; *p*-value = 0.307; *I*^2^ = 13.7%). Studies were divided according to study type, both cohort and case-control study showed a tight association between MetS and CRC risk (Pooled RR = 1.22; 95% CI: 1.20-1.24; and Pooled RR = 1.73; 95% CI: 1.47-1.98, respectively), and with no significant heterogeneity (*p*-value = 0.283, *I*^2^ = 14.3%; and *p*-value = 0.712, *I*^2^ = 0%, respectively). As our data showed, studies conducted in USA and Asia, with publication year ≥ 2012, and with MetS defined by ATP III or IDF showed a more prominent association between MetS and CRC incidence and a good homogeneity.

In terms of CRC mortality (**Figure 4B**), by survival outcome, significance was found in studies investigating cancer-specific survival (CSS) (Pooled RR = 1.72; 95% CI: 1.03-2.42; *p*-value = 0.000; *I*^2^ = 84.6%), but not in the overall survival (OS) (Pooled RR = 1.07; 95% CI: 0.96-1.18; *p*-value = 0.000; *I*^2^ = 85.2%). Studies were analyzed according to average age, and we found that the older people with MetS suffer a higher risk of mortality. A RR of 1.24 (95% CI: 1.18-1.39) was found in male, with a between-study heterogeneity (*p*-value = 0.433; *I*^2^ = 0%), however, only two articles reported specific mortality in female, and a RR of 0.98 (95% CI: 0.64-1.32) was found with a significant heterogeneity (*p*-value = 0.006;* I*^2^ = 86.9%). By study design, the association between MetS and CRC mortality was statistically significant in cohort studies (Pooled RR = 1.26; 95% CI: 1.10-1.41). Since most studies were conducted in Chinese, we split the studies into Chinese by ethnicity, and found that there was no significant correlation between MetS and CRC mortality in Chinese. Articles with MetS defined by ≥3 metabolic abnormalities showed a more prominent association between MetS and CRC incidence. Grouping studies according to TNM stage, risk magnitude did not differ between the two groups.

### Association between individual components of MetS and CRC

The influence of any single component of MetS on CRC incidence and mortality was summarized in **Table 3** and the detailed information was presented in Table S1 and S2. In terms of CRC incidence, both obesity and dysglycemia (high fasting or postprandial glucose, or reported diabetes) are significant risk factors for the incidence of CRC regardless of the sex. Interestingly, both hypertension and hyperlipoidemia were risk factors for the incidence in male but not in female. In terms of CRC mortality, only dysglycemia was a significant risk factor for the mortality of CRC, with significant heterogeneity between studies. Obesity tended to be associated with an increased mortality risk.

### Sensitivity analysis and publication bias

Sensitivity analyses were conducted to examine the stability of the estimates for the accociation between MetS and incidence and mortality of CRC (Figure S1). The sensitivity analysis showed the summary RRs were not markedly changed by any individual study, indicating no significant influence of single study on the results. Non-significant publication bias was found for either of incidence (Kendall's tau = -0.02, *p* = 1.00) or survival (Kendall's tau = 0.71, *p* = 0.48).

## Discussion

This meta-analysis focused on the association of MetS and its components with the incidence and progression of colorectal cancer, involving 18 studies with incidence and 12 studies with survival outcomes, respectively. The results from this meta-analysis indicated that MetS is associated with an increased risk of CRC incidence and mortality. We observed 25% increased cancer incidence, and 15% increased cancer mortality in patients with MetS. In a meta-analysis 55, which discussed the correlation of MetS with digestive tract cancer, no significant association was observed between MetS and CRC mortality. Another meta-analysis on the relationship between MetS and CRC incidence and mortality was performed in 2013 28. In this mentioned meta-analysis, MetS is associated with an increased risk of CRC incidence and mortality both in male and female. However, in this current study, we found no significant correlation between MetS and CRC mortality in female.

In subgroup analysis, the risk estimates of CRC incidence changed little depending on sex, age, cancer site (colon and rectum), type of studies (cohort vs non cohort), ethnicity (Europe, USA, Asia), publication year, or definition of MetS. According to previous studies, MetS may increase the risk of CRC in male rather than female 18, 20. In contrast, according to our meta-analysis, the risk estimates of CRC incidence changed little depending on sex. People with age over 55 years old may suffer more from MetS. In addition, the correlation may also differ because of cancer site 23. However, in this current meta-analysis, there is no significance between the two different cancer sites. A study conducted in South Asians, indicated that definition of MetS by the IDF is the most sensitive in predicting the risk of CRC, compared to MetS as defined by the WHO and ATP III 56. According to our meta-analysis, MetS increased the risk of CRC as defined either IDF or ATPIII, other than defined as AHA.

As for CRC mortality, further stratified analysis indicated statistical significance in studies with assessing cancer-specific survival outcome, in male, a cohort design, ethnicity of non-Chinese or with definition of MetS as ≥ 3 metabolic abnormalities. The risk estimates of CRC mortality changed little depending on age; however, older people with MetS may suffer a higher mortality of CRC. According to our meta-analysis, MetS has no effect on CRC mortality in female. However, an obvious heterogeneity was observed and might decrease the reliability. So, more studies are needed to further clarify this. Since most of the studies were conducted in China, the associations in Europe and USA should be further considered. In addition, most studies identified the overall survival of CRC with MetS, however, the data of cancer-specific survival outcome should be enriched.

Metabolic syndrome (MetS) is a cluster of metabolic risk factors that includes abdominal obesity, hypertension, hyperglycemia, and dyslipidemia, mainly high serum triglyceride and low serum high-density lipoprotein 1. In our meta-analysis, obesity and hyperglycemia are risk factors of CRC incidence in both male and female, which were in consistence with the previous meta-analysis 28. Hypertension and hyperlipoidemia were also indicated risk factors for CRC incidence, especially in male. Since the numbers were too little for statistical analysis, we didn't discuss the difference of sex in low HDL-cholesterol. Experimental studies showed that HDL-cholesterol might promote tumorigenesis through regulation of apoptosis or its influence on cell cycle entry 57, which might explain the role of low HDL-cholesterol in increasing the risk of CRC. Since almost no studies showed the relationship between MetS and CRC mortality in different sex, so we didn't discuss the association according to sex. As our data showed that only dysglycemia is the risk factor for both CRC incidence and mortality. Dysglycemia (high fasting or postprandial glucose, or reported diabetes) may act as carcinogenic agent through promoting the epithelial mesenchymal transition phenomenon 58 and promoting cancer cell proliferation 59. Uptaking of high glucose by cancer cells is associated with advanced grading, greater metastatic potential and cancer chemotherapy resistance 60.

There are potential limitations existing in our study which should be considered. Significant heterogeneity was observed between the studies. Important confounders were not always fully controlled for, which might result in some overestimation of effects due to residual confounding. Studies included used different factors and cut-off points, which complicate comparisons between studies.

In conclusion, MetS is associated with an increased risk of CRC incidence and cancer-specific mortality, but not overall mortality. As for age, the older MetS patients (over 55 years old) are with an increased risk of CRC incidence and mortality. In addition, MetS may increase the CRC mortality in male rather than in female. Moreover, MetS increases the CRC mortality in non-Chinese rather than in Chinese. However, since most of the studies on CRC mortality were conducted in Chinese, further studies are needed to clarify this connection. Among the single components of the syndrome, dysglycemia was the only factor that increased the risk of incidence and mortality of CRC. The pathophysiological mechanisms between MetS and CRC should be further clarified. Our study fully clarified the association of MetS and its components with both CRC incidence and survival. Especially, our results will provide reference in the strategies of CRC prevention and managements.

## Supplementary Material

Supplementary figures and tables.

## Figures and Tables

**Table 1:** Studies reporting on the association of MetS with CRC incidence

Authors, region, design, year	Sex	Age (mean or median or range) y	Cases M/F	Cohort size or controls	Follow-up y	Definition of MetS	RR, 95% CI	Adjustment	QS
Ahmed et al. USA, Cohort, 2006	M/F	45-64	107/87	M: 6630 F: 7563	11.5	≥3 metabolic abnormalities	1.39, 0.90-2.20	Age, sex, exercise, NSAIDs, aspirin use, smoking, alcohol	8
Stürmer et al. USA, Cohort, 2006	M	53.8	494	22046	19	ATP III	M, 1.40, 0.90-2.10	Age, exercise, smoking, alcohol, NSAIDs	7
Bowers et al. Finland, Cohort, 2006	M	59	410	28983	14.1	≥3 metabolic abnormalities	M, 1.40, 1.12-1.74	Age, smoking, TC	8
Russo et al. Italy, Cohort, 2008	M/F	≥40	60/61	16677	2.7	Use of drugs for DM, hypertension, hypercholesterolemia	1.08, 0.90-1.29	NR	6
Stocks et al. Sweden, C/C, 2008	M/F	M: 59.8; F: 59.4	125/181	595 controls	NR	WHO	2.57, 1.20-5.52	Age, sex, blood sample date, fasting time	8
Inoue et al. Japan, Cohort, 2009	M/F	M: 56.5 F: 55.5	155/157	M: 9548 F: 18176	10.2	AHA	M, CC, 1.29, 0.82-2.02 F, CC, 1.03, 0.65-1.65 M, RC, 0.62, 0.29-1.34 F, RC, 0.99, 0.51-1.92	Age, area, smoking, alcohol, TC	9
Pelucchi et al. Italy, C/C, 2010	M/F	31-79	1310/946	4661 controls	NR	IDF	1.69, 1.23-2.33	Age, sex, education, smoking, alcohol, exercise	6
Aleksandrova et al. Europe, C/C, 2011	M/F	CC: 58.8 RC: 58.1	531/562	1093 controls	3.7	ATP III	CC, 1.91, 1.47-2.42 RC, 1.45, 1.02-2.06	Dietary consumption	8
Li et al. China, C/C, 2011	M/F	59	936/570	3354 controls	NR	IDF	1.64, 1.14-2.49	NR	6
Stocks et al. Norway, Austria, Sweden, Cohort, 2011	M/F	M: 43.9 F: 44.1	2834/1861	M: 289866 F: 288834	12	≥3 metabolic abnormalities	M, 1.25, 1.18-1.32 F, 1.14, 1.06-1.22	Age, smoking, components of metabolic abnormalities	8
Kabat et al. USA, Cohort, 2012	F	64.4	81	4862	12	ATP III	F, 2.15, 1.30-3.53	Age, ethnicity, BMI, alcohol, family history, exercise, participation trial, treatment	8
Ulaganathan et al. Malaysia, C/C, 2012	M/F	61.5	80/60	M: 160 controls F: 80 controls	NR	IDF	2.61, 1.53-4.47	Age, sex, ethnic, education, components of MetS, obesity related biomarkers, energy intake, exercise, smoking, alcohol	9
Osaki et al. Japan, Retrospective cohort, 2012	M/F	58.6	98/136	M: 8329 F: 15386	9.1	IDF	M, CC, 1.11, 0.55-2.26 M, RC, 1.84, 0.79-4.27 F, CC, 0.90, 0.49-1.65 F, RC, 1.39, 0.60-3.20	Age, smoking, alcohol, presence of MetS or pre-MetS of each definition	6
Kontou et al. Greece, C/C, 2012	M/F	62	146/93	250 controls	NR	ATP III	1.66, 1.02-2.69	Age, sex, BMI, family history, exercise, smoking	8
Liang et al. USA, Cohort, 2017	F	66.7	114	5068	14.3	ATP III	F, 1.49, 1.02-2.18	Age, ethnicity, smoking, alcohol, exercise, energy intake, dietary fiber, calories percent, family history, NSAIDs, treatment	9
Choi et al. Korea, Cohort, 2018	M/F	54	63045	6296903	5.3	IDF	1.22, 1.20-1.24	Age, sex, smoking, alcohol, exercise	9
Milano et al. Italy, Cross-sectional, 2019	M/F	61	213	5707	NR	AHA	1.92, 1.42-2.58	Age, sex, exercise, component of MetS	7
Li et al. China, Cohort, 2019	M	51.2	394	104333	8.9	AHA	1.22, 0.97-1.53	Age, education, income, smoking, alcohol, sitting time	9

MetS: metabolic syndrome; CRC: Colorectal cancer; BMI: Body mass index; RR: relative ratio; CI: Confidence interval; M: male; F: female; N.R: not reported; NSAIDs: non-steroidal anti-inflammatory drugs; TC: total cholesterol; DM: diabetic mellitus; AHA: American Heart Association; ATP III: Adult Treatment Panel III; C/C: Case-control; IDF: International Diabetes Federation; CC: colon cancer; RC: rectal cancer; WHO: World Health Organization.

**Table 2:** Studies reporting on the association of MetS with CRC mortality

Authors, region, design, year	Sex	Age (mean or median or range) y	Cohort size or controls	Follow-up period, month	Definition of MetS	RR, 95% CI	Adjustment	Stage	Survival	QS
Shen et al. China, Cohort, 2010	M/F	64.1	507	45.1	≥3 metabolic abnormalities	CC, 1.63, 1.04-2.57 RC, 1.94, 1.08-3.50	NR	I-IV	CSS	7
Matthews et al. USA, Cohort, 2010	M	47.2	33230	14.4	ATPIII	1.71, 0.97-3.02	Age, examination year, height, smoking, alcohol, family history, treadmill test duration	I-IV	OS	9
Stocks et al. Europe, Cohort, 2011	M/F	M, 43.9 F, 44.1	M: 2761 F: 1815	12	≥3 metabolic abnormalities	M, 1.25, 1.18-1.32 F, 1.14, 1.06-1.22	Age, smoking, components of metabolic abnormalities	I-IV	OS	8
Yang et al. China, Cohort, 2013	M/F	77.1	36079	72	ATPIII	0.98, 0.93-1.02	Age, sex, race, marital status, education, income, comorbidity, year of diagnosis, stage, grade	I-IV	OS	9
Ahmadi et al. Iran, C/C, 2015	M/F	54	1127	25	NR	0.95, 0.52-1.50	Age, sex, smoking, tumor size, histological type, differentiation, stage, family history, education, alcohol, marital status	I-IV	OS	9
You et al. China, C/C, 2015	M/F	67	1069	59.6	CDS	0.79, 0.59-1.06	Age, sex, stage, differentiation, HDL, uric acid, carcinoembryonie antigen	I-III	OS	9
Cespedes et al. USA, Cohort, 2016	M/F	64	2446	72	AHA	1.23, 1.03-1.56	Age, race, sex, smoking, stage, grade, chemotherapy, radiation, site, sex-specific tertile of muscle tissue at diagnosis	I-III	OS	9
Peng et al. China, Cohort, 2016	M/F	56.4	1318	58.6	CDS	2.98, 2.40-3.69	Age, sex, smoking, alcohol, family history, year of diagnosis, cancer site, stage	I-IV	CSS	9
You et al. China, C/C, 2017	M/F	65.2	1163	71.2	CDS	0.93, 0.83-1.05	Age, sex, platelet to lymphocyte ratio, stage	I-IV	OS	8
Chen et al. China, R/C, 2018	M	50.9	838	40.6	CDS	1.13, 1.06-1.48	NR	I-III	OS	8
Croft et al. Canada, Cohort, 2019	M/F	68.9	142	65.3	NR	1.09, 0.27-4.30	NR	I-III	OS	8
Mafiana et al. Arab, R/C, 2019	M/F	55	301	NA	AHA	1.01, 0.64-1.59	Age, sex, stage, differentiation, cancer treatment, alcohol, smoking	I-IV	CSS	9

MetS: metabolic syndrome; CRC: Colorectal cancer; HDL: high-density lipoprotein; RR: relative ratio; CI: Confidence interval; M: male; F: female; N.R: not reported; C/C: Case-control; R/C: Retrospective cohort; AHA: American Heart Association; ATP III: Adult Treatment Panel III; CDS: Chinese Diabetes Society; CC: colon cancer; RC: rectal cancer; OS: overall survival; CSS: cancer-specific survival.

**Table 3:** Association between individual components of MetS and CRC

Stratification factor	Sex	ES, 95% CI	Heterogeneity, *I*^2^	*p* value
**Incidence**				
Obesity	M+F	1.11, 1.06-1.16	41.5%	0.034
	M	1.12, 1.09-1.16	39.2%	0.117
	F	1.10, 1.00-1.20	56.4%	0.025
Hypertension	M+F	1.04, 1.01-1.06	37.1%	0.058
	M	1.09, 1.06-1.13	0.0%	0.950
	F	0.97, 0.84-1.10	75.8%	0.000
Dysglycemia	M+F	1.14, 1.11-1.17	9.2%	0.345
	M	1.16, 1.11-1.20	22.9%	0.247
	F	1.21, 1.17-1.26	0.0%	0.782
Hyperlipoidemia	M+F	1.00, 0.94-1.06	60.1%	0.001
	M	1.10, 1.05-1.15	48.9%	0.057
	F	1.03, 0.99-1.07	41.7%	0.100
Low HDL-C	M+F	1.04, 0.87-1.21	57.3%	0.016
**Mortality**				
Obesity	M+F	1.04, 0.97-1.12	59.7%	0.015
Hypertension	M+F	1.01, 0.91-1.11	66.0%	0.003
Dysglycemia	M+F	1.10, 1.01-1.20	59.1%	0.007
Hyperlipoidemia	M+F	1.04, 0.91-1.18	79.3%	0.000

ES: effect size; 95% CI: 95% confidence interval; M: male; F: female.

## Abbreviations

MetS: Metabolic syndrome
CRC: Colorectal cancer
CIs: Confidences Intervals
NOS: Newcastle Ottawa Scale
RCTs: randomized controlled trials
PRISM: preferred reporting items for systematic reviews and meta-analyses
BMI: Body mass index
RR: relative ratio
M: male
F: female
N.R: not reported
NSAIDs: non-steroidal anti-inflammatory drugs
TC: total cholesterol
DM: diabetic mellitus
AHA: American Heart Association
ATP III: Adult Treatment Panel III
C/C: Case-control
IDF: International Diabetes Federation
WHO: World Health Organization
HDL: high-density lipoprotein
R/C: Retrospective cohort
CDS: Chinese Diabetes Society
OS: overall survival
CSS: cancer-specific survival
ES: effect size
