# Supplementary material for: The association of Metabolic Syndrome and its Components with the Incidence and Survival of Colorectal Cancer: A Systematic Review and Meta-analysis
Source: Int J Biol Sci. 2021 Jan 1;17(2):487–97. doi: 10.7150/ijbs.52452 (PMC7893592; doi:10.7150/ijbs.52452)
Supplement: Supplementary file 1 — Supplementary figures and tables. [file ijbsv17p0487s1.pdf]

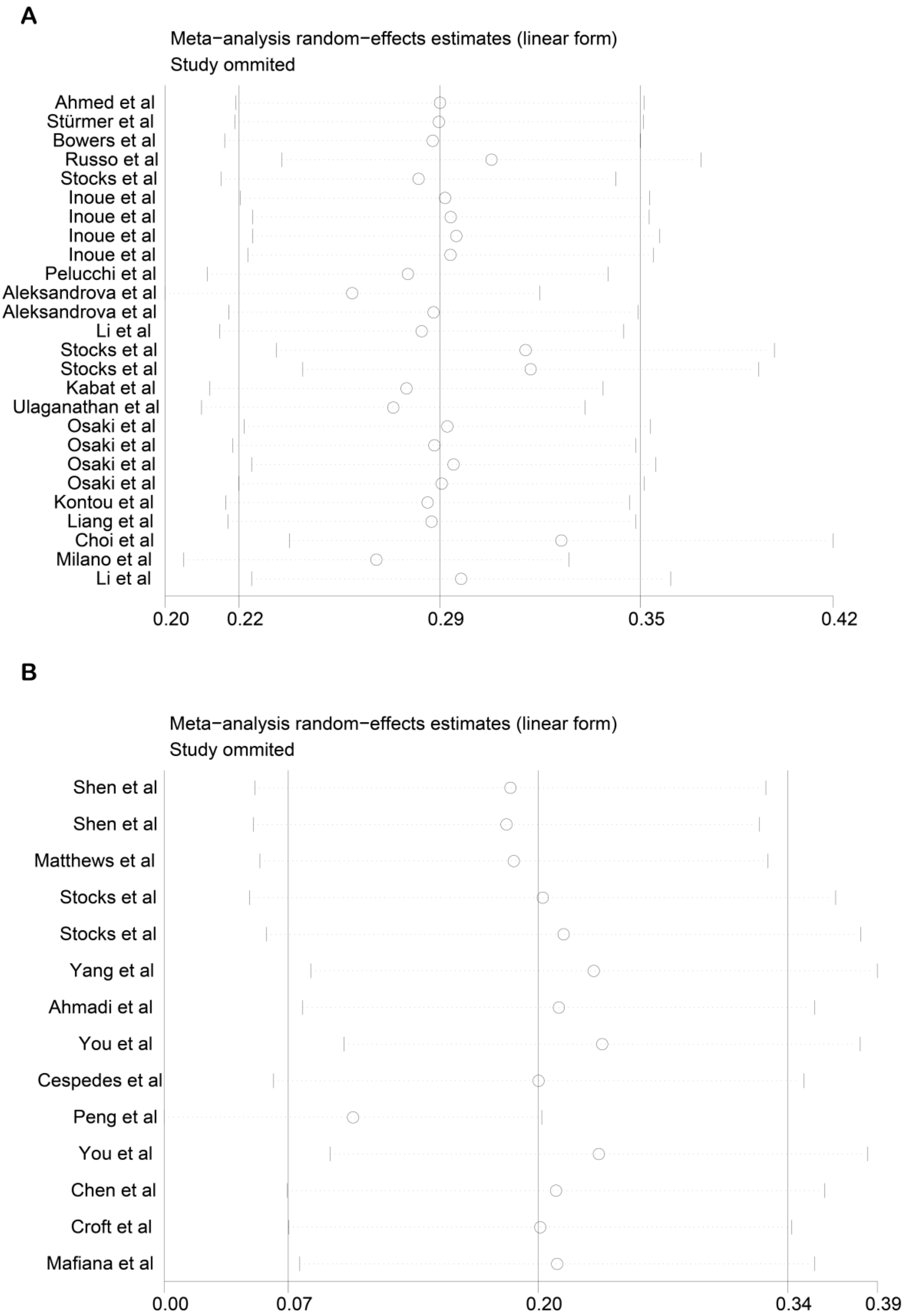

Figure S1 Sensitivity Analysis for the effect of MetS on incidence and mortality of CRC. (A) Sensitivity Analysis for the effect of MetS on CRC incidence. (B) Sensitivity Analysis for the effect of MetS on CRC mortality.

Table S1 Influence of any single component and MetS on CRC incidence

Study	Stratification factor	Obesity	Hypertension	Dysglycemia	Hyperlipoidemia	Low HDL-C
Ahmed et al	CRC, M+F	1.40 (1.00-1.90)	1.35 (1.00-1.80)	1.39 (1.00-1.80)	1.08 (0.80-1.50)	1.19 (0.9-1.6)
Stürmer et al	CRC, M+F	1.40 (1.10-1.70)	1.10 (0.90-1.30)	1.50 (1.10-2.00)	0.90 (0.07-1.10)	NR
Stocks et al	CRC, M+F	1.79 (1.09-2.95)	1.49 (0.90-2.49)	2.03 (0.86-4.79)	NR	NR
Inoue et al	CC, M	0.80 (0.50-1.28)	0.99 (0.64-1.52)	0.83 (0.51-1.34)	1.71 (1.11-2.62)	1.15 (0.65-2.03)
Inoue et al	CC, F	1.04 (0.69-1.57)	1.07 (0.71-1.62)	0.90 (0.50-1.64)	1.00 (0.63-1.60)	0.41 (0.14-1.16)
Inoue et al	RC, M	1.53 (0.85-2.75)	1.18 (0.65-2.15)	0.81 (0.40-1.64)	0.54 (0.26-1.11)	1.12 (0.74-1.71)
Inoue et al	RC, F	0.92 (0.50-1.68)	0.60 (0.34-1.06)	1.35 (0.62-2.92)	0.52 (0.24-1.13)	1.14 (0.63-2.06)
Pelucchi et al	CRC, M	1.26 (1.08-1.48)	1.24 (1.03-1.48)	1.27 (0.95-1.69)	1.14 (0.93-1.40)	NR
Pelucchi et al	CRC, F	1.06 (0.86-1.30)	0.87 (0.71-1.06)	1.20 (0.82-1.75)	0.83 (0.66-1.03)	NR
Li et al	CRC, M+F	1.10 (0.93-1.32)	1.08 (0.88-1.29)	1.14 (0.95-1.40)	1.03 (0.83-1.25)	NR
Stocks et al	CC, M	1.10 (1.03-1.17)	1.10 (1.01-1.21)	0.98 (0.82-1.17)	1.01 (0.92-1.10)	NR
Stocks et al	RC, M	1.04 (0.95-1.13)	1.10 (0.95-1.23)	1.08 (0.87-1.34)	1.01 (0.90-1.12)	NR
Stocks et al	CC, F	1.13 (1.05-1.22)	0.91 (0.81-1.02)	1.08 (0.89-1.32)	1.07 (0.98-1.18)	NR
Stocks et al	RC, F	0.97 (0.86-1.09)	0.99 (0.84-1.17)	1.22 (0.92-1.62)	1.11 (0.97-1.28)	NR
Kabat et al	CRC, F	1.08 (0.50-2.34)	1.82 (0.98-3.38)	1.79 (0.99-3.24)	1.33 (0.76-2.32)	1.22 (0.67-2.25)
Choi et al	CRC, M+F	1.16 (1.12-1.20)	1.04 (1.02-1.07)	1.15 (1.12-1.18)	0.93 (0.90-0.97)	1.15 (1.10-1.19)
Milano et al	CRC, M+F	1.06 (0.92-1.23)	1.10 (0.94-1.29)	1.00 (0.86-1.15)	0.92 (0.79-1.08)	0.84 (0.72-1.08)
Li et al	CRC, M	1.32 (1.07–1.64)	1.04 (0.82–1.31)	1.19 (0.96–1.48)	1.12 (0.90–1.41)	1.24 (0.84–1.84)

M, male; F, female; CRC: Colorectal cancer; HDL: high-density lipoprotein; CC: colon cancer; RC: rectal cancer; N.R: not reported

Table S2 Influence of any single component and MetS on CRC mortality

Author	Sex	Obesity	Hypertension	Dysglycemia	Hyperlipoidemia
Stocks et al	M	1.07 (1.02-1.13)	1.10 (1.02-1.18)	1.02 (0.89-1.17)	1.17 (1.06-1.28)
Stocks et al	F	1.08 (1.01-1.15)	0.94 (0.85-1.03)	1.12 (0.95-1.32)	1.02 (0.91-1.15)
Yang et al	M+F	0.99 (0.91-1.07)	1.08 (1.03-1.12)	1.17 (1.13-1.21)	0.78 (0.70-1.08)
Ahmadi et al	M+F	0.62 (0.44-8.70)	0.83 (0.42-1.64)	1.45 (0.71-2.95)	NR
You et al	M+F	0.89 (0.66-1.19)	0.90 (0.69-1.19)	0.97 (0.63-1.48)	0.97 (0.85-1.11)
Cespedes et al	M+F	1.25 (1.00-1.56)	0.84 (0.68-1.03)	1.07 (0.90-1.26)	1.23 (1.03-1.56)
You et al	M+F	0.99 (0.95-1.02)	0.86 (0.70-1.06)	1.00 (0.85-1.17)	0.95(0.84-1.07)
Chen et al	M+F	0.96 (0.81-1.10)	1.12 (0.91-1.34)	1.01 (0.93-1.09)	1.01 (0.86–1.15)
Croft et al	M+F	NR	3.10 (0.83-12.22)	1.32 (0.51-3.40)	1.86 (0.58-5.97)

M, male; F, female; N.R: not reported
